# Behavior of Metalloproteinases in Adipose Tissue, Liver and Arterial Wall: An Update of Extracellular Matrix Remodeling

**DOI:** 10.3390/cells8020158

**Published:** 2019-02-14

**Authors:** Gabriela Berg, Magalí Barchuk, Verónica Miksztowicz

**Affiliations:** 1 Laboratorio de Lípidos y Aterosclerosis, Departamento de Bioquímica Clínica, Facultad de Farmacia y Bioquímica, Universidad de Buenos Aires, Buenos Aires 1113, Argentina; magalibarchuk@gmail.com (M.B.); veronicajmik@gmail.com (V.M.); 2 Facultad de Farmacia y Bioquímica, Instituto de Fisiopatología y Bioquímica Clínica (INFIBIOC), Universidad de Buenos Aires, Buenos Aires 1113, Argentina; 3 Facultad de Farmacia y Bioquímica, CONICET, Universidad de Buenos Aires, Buenos Aires C1425FQB, Argentina

**Keywords:** extracellular matrix, metalloproteinases, adipose tissue, liver, arterial wall

## Abstract

Extracellular matrix (ECM) remodeling is required for many physiological and pathological processes. Metalloproteinases (MMPs) are endopeptidases which are able to degrade different components of the ECM and nucleus matrix and to cleave numerous non-ECM proteins. Among pathological processes, MMPs are involved in adipose tissue expansion, liver fibrosis, and atherosclerotic plaque development and vulnerability. The expression and the activity of these enzymes are regulated by different hormones and growth factors, such as insulin, leptin, and adiponectin. The controversial results reported up to this moment regarding MMPs behavior in ECM biology could be consequence of the different expression patterns among species and the stage of the studied pathology. The aim of the present review was to update the knowledge of the role of MMPs and its inhibitors in ECM remodeling in high incidence pathologies such as obesity, liver fibrosis, and cardiovascular disease.

## 1. Introduction

The extracellular matrix (ECM) is a multimolecular complex structure comprising collagen and elastin fibers, structural glycoproteins including fibronectin and laminin, and mucopolysaccharides. It is organized into a three-dimensional (3D) network, and in physiological states, a balance between synthesis, deposit and degradation of ECM components exists [1]. ECM provides the structural framework to cells. The composition of ECM varies among multicellular structures, with the fibroblasts and epithelial cells being the most common cell types in the stromal architecture. The local components and composition of ECM collectively determine the biochemical properties of the connective tissue [2]. Different pathological processes, such as adipose tissue (AT) expansion, fibrosis, and atherosclerotic plaque development and rupture are characterized by ECM remodeling, in which many regulator factors and proteolytic enzymes are involved. Metalloproteinases (MMPs) are the main actors involved in ECM degradation, and the regulation of MMPs expression and activity is crucial for tissues homeostasis. Changes in the MMPs pattern expression or in the balance between MMPs and their tissue-specific inhibitors alters ECM biology. The aim of the present review was to update the knowledge of the role of MMPs and its inhibitors in ECM remodeling in high incidence pathologies such as obesity, liver fibrosis, and cardiovascular disease.

## 2. Metalloproteinases Characteristics

Metalloproteinases (MMPs) constitute a family of zinc-calcium dependent endopeptidases able to degrade different components of ECM and to cleave numerous non-ECM proteins, such as adhesion molecules, cytokines, protease inhibitors, and membrane receptors. Structurally, MMPs are constituted by a N-terminal propeptide domain, a cysteine-containing switch motif Zn^2+^-containing conservative catalytic domain, a C-terminal proline-rich hinge region, and a hemopexin domain [3]. 

**Figure 1:**
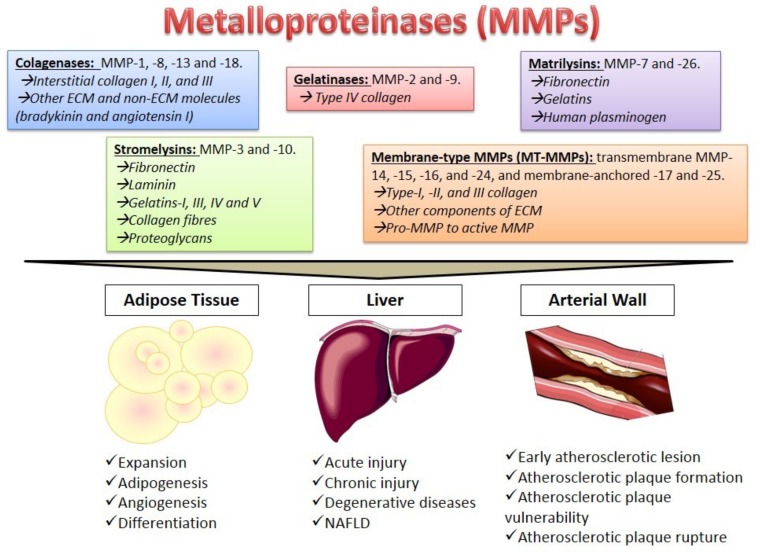
The metalloproteinases family and their main actions in adipose tissue, the liver, and the arterial wall. Arrows and italics represent Metalloproteinases (MMPs) substrates.

So far, 28 MMPs are known which are classified according to their substrate specificity in the following: Collagenases (MMP-1, MMP-8, MMP-13 and MMP-18), which cleave interstitial collagen I, II, and III and also other ECM and non-ECM molecules, such as bradykinin and angiotensin I; gelatinases (MMP-2 and MMP-9), mainly responsible for type IV collagen degradation; stromelysins (MMP-3 and MMP-10), which degrade fibronectin, laminin, gelatins-I, -III, -IV and -V, collagen fibers and proteoglycans; matrilysins (MMP-7 and MMP-26), responsible for fibronectin, gelatins, and human plasminogen hydrolysis [4]. In addition, membrane-type MMPs (MT-MMPs), including transmembrane MMP-14, MMP-15, MMP-16 and MMP-24, and membrane-anchored MMP-17 and MMP-25 have been defined. MT-MMPs can degrade type-I, -II, and -III collagen and other components of ECM, and pro-MMP to active MMP. Finally, there are some non-classified MMPs, such as MMP-12, MMP-19, MMP-20, MMP-21, MMP-23, and MMP-28, in which expression is often tissue-specific [4] (Figure 1).

According to the MMPs functions mentioned above, these enzymes play important roles in physiological processes such cell proliferation, angiogenesis, and wound healing, among others; However, they are also involved in pathological processes including AT expansion, liver fibrosis and atherosclerosis [5] (Figure 1).

Besides the well-known role of MMPs in ECM remodeling, these enzymes are important in numerous nuclear events. It has been demonstrated that many MMPs have a nuclear localization signaling sequence that allows them to translocate into the nucleus after activation and regulate several mechanisms such as the degradation of nuclear proteins and transcription regulation [6]. Different studies suggest that nuclear MMPs could induce apoptosis in cardiac myocytes, endothelial cells [7], and renal tubular cells [8]. Moreover, some nuclear MMPs can bind directly to DNA promoters and regulate the transcription of multiple genes, such as heat shock family proteins and different growth factors [6]. 

## 3. MMPs Regulation

**Figure 2:**
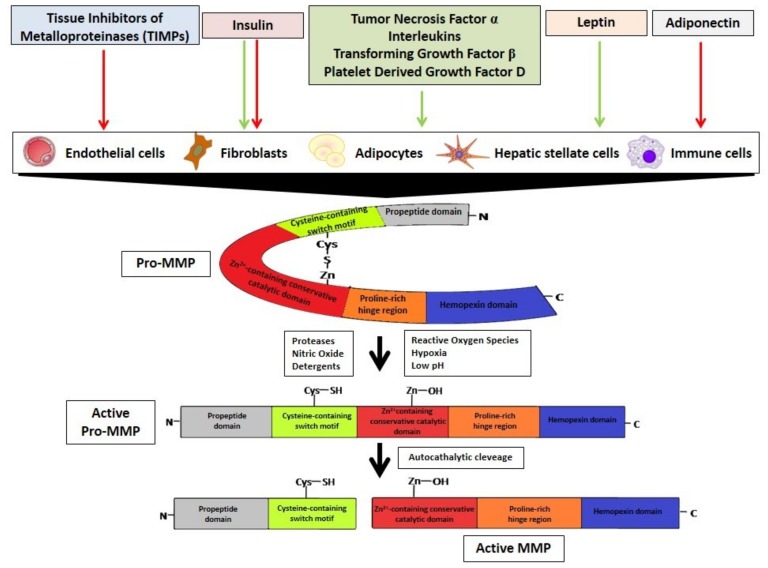
The activation of Metalloproteinases. MMPs are secreted by different cell types as inactive Pro-MMPs. These Pro-MMPs could be activated by different factors via the hydrolysis of the Cys-S-Zn bond. The active Pro-MMPs undergo auto-cleavage to generate the active MMPs. Many hormones regulate MMPs production by inhibiting and/or activating it. The green arrows indicate activation; red arrows indicate inhibition.

The MMPs are regulated at different levels: Gene expression, proteolytic activation of the proenzymes, inhibition of the catalytic activity by chemical and biological agents, and complexing with specific tissue inhibitors (TIMPs). MMPs are synthesized by different cells types such as endothelial cells, fibroblasts, adipocytes, hepatic stellate cells (HSC), and immune cells (Figure 2). In physiological conditions, they are expressed at some baseline levels but are differentially expressed in response to certain hormones, growth factors, and inflammatory and fibrogenic cytokines [9], via the activation of MAPK, JNK, and NF-κB-dependent signaling pathways [10]. 

MMPs are secreted as latent zymogens (pro-MMPs) which are inactive due to the interaction of the zinc ion in the catalytic domain with the sulfhydryl group of the cysteine residue in the prodomain. Pro-MMPs are activated by the disruption of the cysteine switch by different mechanisms such as the proteolytic action of other proteases, conformational changes generated by nitric oxide (NO), reactive oxygen species (ROS) and hypoxia processes, or by chaotropic agents and denaturants such as sodium dodecyl sulfate and low pH and heat treatment, among others. In all cases the -SH group is replaced by H_2_O and the enzyme gains the ability to hydrolyze the propeptide for its complete activation [11] (Figure 2).

Finally, MMPs can be inhibited by TIMPs. Four TIMPs (TIMP-1, TIMP-2, TIMP-3, and TIMP-4) have been reported which form a complex in MMP catalytic domains in a 1:1 stoichiometric ratio. TIMPs have a 2-domain structure in which the N-terminal domain contains the inhibitory residues [12]. TIMPs inhibit most MMPs, except those from TIMP-1, which has a weak inhibitory effect only on MT1-MMP, MT3-MMP, MT5-MMP, and MMP-19 [12]. The local balance between activated MMPs and TIMPs determines the net result of MMPs activity in tissues. In pathological situations, this balance might be disrupted leading to the uncontrolled activation of the MMPs [13].

As mentioned above, MMPs expression is regulated by different factors. Insulin or insulin-like growth factor-1 signaling, through the PI3K/Akt cascade, regulates MMPs in different ways according to the target organ. In animal models, it has been demonstrated that insulin promotes the activation of gelatinases and MT1-MMP through the increase of pro-inflammatory cytokines production [14]. However, hyperinsulinemia showed a contrary effect in the liver, decreasing the expression of the MMP-2, MMP-9, and MT1-MMP [15].

Leptin and adiponectin are adipocytokines synthetized by AT, which also modulate MMPs expression in different manners. Leptin has central and peripheral effects on regulating food intake and energy expenditure, and it is involved in ECM remodeling by regulating the expression of MMPs and TIMPs in many tissues. In vitro studies have reported that leptin promotes MMP-2 secretion by 3T3-L1 preadipocytes [16]. Schram et al. [17] reported that incubation of rat cardiac myofibroblasts with leptin significantly stimulated MT1-MMP expression, resulting in an increase in MMP-2 activity, without changes in protein levels. More recently, studies in vitro and in vivo demonstrated that leptin induces MMP-9 expression in smooth muscle cells [18]. Liberale et al. [19] studied serum levels of leptin and MMP-8 in morbidly obese type 2 diabetes mellitus patients before and after bariatric surgery, showing a significant decrease in both proteins after surgery. The authors concluded that bariatric surgery would be associated with an acute abrogation of leptin affecting MMP-8 levels.

Adiponectin is an anti-inflammatory and anti-atherogenic adipocytokine, inversely associated with expanded AT. Beneficial effects of adiponectin on the vascular wall and atherosclerotic plaque have been further studied. In vitro studies have reported that adiponectin inhibits foam cell formation by increasing NO levels and reducing class A scavenger receptor expression in macrophages [20]. In reference to MMPs, it has been reported that adiponectin would decrease MMPs activity through the increase of TIMPs expression and secretion in human monocyte-derived macrophages via the Syk pathway [21]. In patients, Miksztowicz et al. [22] reported an inverse association between plasma adiponectin levels and circulating activity of MMP-2 in patients with insulin resistance. In accordance, Kou et al. [23] recently demonstrated that adiponectin levels inversely correlate with circulating MMP-9 levels in non-diabetic hypertensive patients. 

## 4. The Role of MMPs in Different Tissues

### 4.1. Adipose Tissue

The expansion of AT is associated with adipogenesis and angiogenesis [24] and different studies have demonstrated that MMPs are involved in both processes. AT is constituted by different cell types (adipocytes, preadipocytes, fibroblasts, endothelial cells, and immune cells) which can be sources of MMPs. Until now, there are several studies about MMPs behavior in AT, still with controversial results, probably due to the different pattern expression that these enzymes present according to the experimental model and the AT distribution.

In abdominal AT from obese animal models, an increase of MMP-3, MMP-11, MMP-12, MMP-13 and MMP-14 levels with decreased expression of MMP-7, MMP-9, MMP-16, MMP-24 and TIMP-4 [25] has been reported. In our laboratory, we also investigated the behavior of MMPs in visceral AT from an animal model of obesity induced by a high-fat diet. In this model, an increase of MMP-9 activity was observed, inversely associated with PPARγ levels, without differences in MMP-2 [26]. In contrast, a decrease in MMP-2 and MMP-9 activity in AT from an animal model of early insulin-resistance (IR) induced by a sucrose-rich diet, without changes in MMPs plasma activity, has been reported [27]. Furthermore, studies in vitro in 3T3-F442A preadipocytes demonstrated that MMPs and TIMPs are expressed with different patterns during the adipogenesis process, with some discrepancies in comparison to in vivo models [25]. MMP-2, MMP-9, MMP-11, and MMP-16 and TIMP-2 mRNAs were expressed in the in vitro differentiated adipocytes; however, they were not detected in isolated mature adipocytes from fat pads obtained from obese mice. These differences could be due to the uncompleted differentiation observed in vitro. Indeed, although a homogenous population of mature adipocytes was isolated from fat pads, in vitro adipogenesis generated a mixture of cells at different stages of differentiation. Moreover, adipocytes generated in vitro displayed a multilocular morphology that differed from the unilocular adipocytes observed in vivo [25].

In obesity, hypoxia and inflammation occur as a consequence of AT expansion, leading to an increase of proinflammatory adipocytokines secretion. As previously mentioned, adipocytokines are involved in the regulation of MMPs expression. In vitro studies have demonstrated that MMP-2 secretion was significantly promoted by leptin treatment in 3T3-L1 preadipocytes [16]. Little is known about the direct effect of adiponectin on MMPs from AT. It has been suggested that adiponectin could directly affect the balance of MMP/TIMP expression in macrophages from AT [13]; however, further studies are necessary to elucidate the role of this cytokine in MMPs behavior in this tissue.

Obesity is also characterized by collagen deposition in AT. Song et al. [28] studied the association between Toll-like receptor (TLR)-2 and collagen I in AT from TLR-2 knockout mice fed a high-fat diet. The authors reported decreased levels of collagen I and TIMP-1 with increased levels of MMP-1 [28], suggesting that TLR-2 would be involved in collagen I metabolism and would exhibit a role in MMP-1 and TIMP-1 behavior in AT from obese mice. In AT, adipose-derived mesenchymal stem cells (AMSCs) are also responsible for the remodeling of three-dimensional ECM barriers during differentiation. However, the molecular mechanism is still not completely described. Almalki et al. [29] recently studied in vitro the expression of MMPs during the differentiation of AMSCs isolated from porcine abdominal AT to endothelial cells [29]. The authors demonstrated that the up-regulation of MMP-2 and MMP-14 has an inhibitory effect on the differentiation of AMSCs to endothelial cells; the silencing these MMPs inhibits the cleavage of the VEGF-receptor and stimulates the differentiation of AMSCs to endothelial cells and consequently the formation of endothelial tubes. These findings provide a potential mechanism for the regulatory roles of MMP-2 and MMP-14 in the re-endothelialization of coronary arteries. 

MMPs have also been studied in human AT. Gummesson et al. [30] studied the gene expression of MMP-9 in subcutaneous AT and plasma in men with and without metabolic syndrome treated with a weight-reducing diet. The authors found a lack of association between AT mRNA and plasma levels of MMP-9, suggesting that this tissue is not a major contributor to circulating MMP-9. Nowadays, one of the main treatments for obesity is bariatric surgery (BS). The impact of BS on MMPs is controversial. It has been reported that in morbidly obese patients, serum MMP-2 and MMP-9 levels significantly decrease after BS [31]. Otherwise, in diabetic obese patients, serum MMP-7 levels remained unchanged after BS [32]. In obese nondiabetic patients, Liu et al. [33] reported an increase in degradation of collagen I and III in subcutaneous AT 1 year after BS, accompanied by increased MMP-2 and MMP-9 activity; however, these differences were not observed in obese diabetic patients. Further studies are needed to explore the behavior and the balance between MMPs and TIMPs in the context of AT remodeling after BS. 

In the last few years, attention has been focused on epicardial adipose tissue (EAT), a visceral AT, which surrounds the myocardium and coronary arteries. Given that there are no fascial boundaries between EAT and heart, this AT has a direct impact on coronaries and myocardium. Different studies have demonstrated that expanded EAT would be associated with coronary artery disease (CAD) [34,35]. The expansion of EAT in CAD requires the plasticity of ECM, in which increased MMPs activity could be involved. Recently, we reported an increase in MMP-2 and MMP-9 activity in EAT from CAD patients compared to patients with no CAD, accompanied by an augmented vascular density [36]. MMPs are essential during the angiogenic process, and even more during the ECM degradation [37]. In EAT from CAD patients, we also reported the association between MMPs activity and vascular endothelial growth (VEGF) levels [36]. Qorri et al. [38] demonstrated that MMP-9 releases VEGF bound to proteoglycans within ECM, enhancing the angiogenesis process. In EAT, MMP-2, and MMP-9 were located mainly in perivascular connective stroma and in the basement membrane surrounding the adipocytes [36]. These results suggest that both MMPs would be partially responsible for the ECM remodeling and the major vascular density necessary for EAT expansion in CAD patients. 

Different studies have demonstrated that expanded EAT in CAD patients is infiltrated by inflammatory immune cells, mainly macrophages and lymphocytes [36,39]. In our laboratory, to determine the macrophages’ polarization to the proinflammatory (M1) or the anti-inflammatory (M2) phenotype, M1 and M2 markers were evaluated. In EAT from CAD patients, an increase of M1 markers was observed, associated with elevated levels of MMP-1, an indicator of activated M1 macrophages (data not published). These results would confirm the observed inflammatory profile of EAT in CAD patients [35,36]. 

### 4.2. The Liver

Acute and chronic liver diseases are characterized by ECM remodeling, which is necessary for the fibrogenesis observed in these pathologies. The most abundant protein within ECM is collagen, of which types I, III, and V are predominant in healthy livers, whereas type IV collagen and laminin are essential constituents of basement membranes. The dysregulation of ECM homeostasis is often associated with degenerative diseases [40]. Depending on the nature of the liver injury, the normal composition of ECM in the space of Disse, the Glisson’s capsule, around portal tracts, and around the central veins is replaced by up to tenfold the amount of type I and type III collagen during fibrogenesis [41]. In healthy livers, HSCs, localized in the perisinusoidal space, are the most important source of ECM. The maintenance of the dynamic structure and composition of the ECM depends on an exactly regulated, moderate turn-over directed by MMPs [42]. Many MMPs are absent or constitutively expressed in healthy liver, but after hepatic injury, their expression increases and probably mediates the ECM breakdown and control of cellular functions. TIMP-1 and TIMP-2 are secreted by HSCs and are abundantly expressed in the liver. Studies in animal models of liver fibrosis suggest that an imbalance between TIMPs and MMPs leads to fibrogenesis [43]. 

Until now, the nature of the behavior of MMPs in the liver fibrosis process has been controversial. Different factors could lead to acute liver injuries, such as toxic xenobiotic metabolites and hepatotrophic viruses producing apoptosis and necrosis of hepatocytes. Beside the role of HSC in secreting ECM components, these cells are also the principal source of acute liver injury-associated gene expression and the early state activation of MMPs. Different studies have demonstrated that, directly after liver injury and previous to fibrogenesis, an increase in some MMPs expression occurs [42,44]. Injured hepatocytes release ROS and pro-inflammatory cytokines with a capacity of activated HSCs which proliferate and secrete ECM components contributing to liver fibrosis. It has been suggested that MMPs activation in the space of Disse would be crucial for the fibrotic activation of HSC in ECM and in liver fibrogenesis [45]. Insulin-like growth factor binding protein-related protein 1 (IGFBPrP1) has been proposed as a novel mediator of hepatic fibrogenesis. Recently, Ren et al. [46] reported that hepatic expression of IGFBPrP1 was increased in an animal model with induced hepatic fibrosis, associated with a concomitant MMP2/TIMP2 and MMP9/TIMP1 imbalance. Furthermore, the IGFBPrP1 knockdown attenuated liver fibrosis by re-establishing the MMP2/TIMP2 and MMP9/TIMP1 balance concomitant with the inhibition of HSCs activation and degradation of the ECM [46]. 

In reference to liver chronic damage, non-alcoholic fatty liver disease (NAFLD) is the most prevalent chronic hepatic disease in the world, and it is considered to be the hepatic manifestation of metabolic syndrome. One-third of patients with NAFLD progress to liver fibrosis within 4–5 years after the first liver biopsy. The increased levels of pro-inflammatory adipocytokines, characteristic of NAFLD, could be partially responsible for liver damage through the alteration of MMPs and TIMPs balance, leading to enhanced matrix deposition by HSCs and diminished breakdown of connective tissue proteins. Toyoda et al. [47] reported that mRNA and serum levels of MMP-2 were up-regulated in hepatic steatosis; however, Miele et al. [48] found only increased TIMP-1 serum levels. In our laboratory, we recently described a decrease in liver MMP-2 activity in severe fibrosis in comparison to non- to moderate fibrosis stages and no differences in MMP-9 activity in patients with NAFLD [49]. In accordance, Hemman et al. [50] previously reported that in activated HSCs, TIMP-1 expression is increased, and concomitantly MMPs activity is reduced, leading to protein accumulation. Recently, an increase of pro-MMP-2 accompanied by increased TIMP-1 and TIMP-2 levels in NAFLD liver fibrosis [51] has been reported. In accordance, Yilmaz et al. [52] described an increase of TIMP-1 serum levels in patients with significant fibrosis, and this was identified as an independent predictor of histological fibrosis. 

Different antidiabetic drugs have shown antifibrotic properties in the liver. In animal models, it has been reported that treatment with metformin protects against hepatic fibrosis by decreasing the expression of profibrogenic biomarkers such as TIMP-1 [53]. Furthermore, in an experimental liver fibrosis model, a dipeptidyl peptidase-4 inhibitor (DPP4-I), sitagliptin, attenuated liver fibrosis development along with the suppression of hepatic transforming growth factor (TGF)-β1, total collagen, and TIMP-1 levels in a dose-dependent manner [54]. These suppressive effects occurred almost concurrently with the attenuation of HSCs activation [54]. These results suggest therapeutic potential for antidiabetic drugs in humans with liver fibrosis.

### 4.3. The Arterial Wall

It has been proposed that MMPs participate in atherosclerotic plaque development and vulnerability [55] due to their role in ECM degradation. Numerous studies in cell culture, animal models, and humans have deepened our understanding of the behavior of these enzymes and inhibitors in the arterial wall. It is well known that the sentinel cause of plaque formation is the lipid deposition in the intima promoting foam cell development. Modified lipoproteins such as small dense LDL, oxidized LDL (ox-LDL), and remnants of lipoproteins are mainly taken up by macrophages via scavenger receptors (SR), with particularly the type A (SR-A) and CD36 contributing to early foam cell formation and atherosclerotic plaque progression [56]. At the same time, activated vascular smooth muscle cells (VSMC) migrate to the intimal layer and proliferate, producing ECM components. Subsequently, intimal growth is mediated by an increase in the number and diversity of cells and by the accumulation of new ECM for the development and maintenance of the fibrous cap. Ox-LDL promotes MMPs expression through different pathways. The exposure of human macrophages to ox-LDL up-regulates MMP-1 and MMP-14 via NF-κB [57]. These MMPs could participate in the early atherosclerotic plaque development because they are found in early fatty streak lesions [58]. Lectin-like oxidized low-density lipoprotein receptor-1 (LOX-1) is the major ox-LDL receptor in ECs [59], VSMC, and macrophages [60].

Studies in HUVEC demonstrated that activated expression of LOX-1 with ox-LDL up-regulates inflammatory proteins and MMP-2 and MMP-9 expression [61]. On the other hand, MMP-2, MMP-9, and MMP-14 promote VSMC migration and proliferation, which could increase fibrous cap thickness and promote plaque stability [62]. Johnson et al. [63] found that stimulation of macrophages with ox-LDL did not affect MMP-14 and TIMP-3 mRNA expression. However, they observed that the later polarization of macrophages to the M1 phenotype increased mRNA expression of MMP-14 and decreased mRNA levels of TIMP-3, leading to heightened invasive capability, increased proliferation, and augmented susceptibility to apoptosis. In contrast, the macrophages’ polarization to the anti-inflammatory type M2 produced the opposite effect. The fact that foam cells expressed high MMP-14 and less TIMP-3 protein could implicate epigenetic mechanisms [63].

Within the early atherosclerotic lesions, due to the progressive accumulation of lipids and inflammatory cells, fatty streaks tend to progress to advanced lesions, containing lipid droplets, foam cells, macrophages, and lymphocytes. These inflammatory cells have crucial roles in atherosclerosis progression, expressing and releasing different cytokines, which mediate the chemotaxis of additional macrophages, as well as B and T cells [64], raising aberrant inflammatory responses and fostering an inflammatory milieu. In this environment, the high-level expression of MMPs contributes to plaque instability. TNF-α, IL-1β, and CD40L stimulate MMP expression in both monocytes and macrophages [64]. As described in AT, in isolated human plaque-derived cells, Monaco et al. [65] verified that activation of TLR-2 was directly implicated in MMP-1 and MMP-3 production. These inflammatory mediators share the ability to activate the MAPKs, extracellular signal-related kinases 1/2 (ERK1/2), p38 MAPK, and c-jun N-terminal kinase (JNK), as well as phosphoinositide-3 kinase (PI3 kinase) and the inhibitor of κB kinase-2 (IKK2) that leads to the activation of NF-κB [64], which may account for the upregulation of MMPs [66]. More recently, in an apolipoprotein E (ApoE) knockout model with mice fed a high-fat diet, it was demonstrated that T-box21 (T-bet) knockout has no effect on M1 macrophage polarization and only marginally increases M2 polarization. Moreover, T-bet knockout does not reduce and may even increase the mRNA expression of several MMPs that have been shown to promote plaque development [67]. The chronic state of hypoxia found in advanced atherosclerotic plaque increases MMP-7 expression [68], and transcriptomic data from hypoxic macrophages indicates that MMP-1, MMP-3, MMP-10, and MMP-12 are also significantly upregulated, possibly due to increased production of IL-1α,β [69]. Moreover, hypoxia-inducible factor-1α and hypoxia-inducible factor-2α [70] and JAK2/STAT-3 pathways have been implicated in the overexpression of these MMPs [71].

In contrast, the overexpression of suppressors of cytokine signaling (SOCS) proteins inhibits MMP-1 and MMP-9 expression in monocytes and macrophages. PPARα selectively inhibits MMP-12 production by directly binding to components of the AP-1 complex [72], whereas PPARα and PPARγ inhibit MMP-9 secretion from human macrophages [64]. Herein the role of PPAR agonists as potential therapeutic options must be highlighted.

Nevertheless, given the significant differences in the MMP mRNAs expression pattern and in the morphological characteristics of the atherosclerotic plaques among species, the extrapolation from animal models to humans is discouraging. Mice and human regulate a different spectrum of proteinases, including MMPs, during physiological responses [55]. So far, studies of genome-wide association (GWAS) in humans have been important. These studies provided evidence of the pathogenic role of MMP-12, in which gen was significantly overexpressed in carotid plaques compared to atherosclerosis-free control arteries [73]. GWAS studies also indicated that genetic variation determines a significant portion of circulating MMP-8 concentrations associated with cardiovascular disease [74]. Biobank studies have also demonstrated an association between MMP-8, -9 and -12 with plaque morphologies, suggesting vulnerability to rupture [64]. Further modern studies involving Mendelian randomization should consider MMPs association with plaque rupture and cardiovascular disease. 

Statins are known to exert stabilizing effects on atherosclerotic plaque. Studies in vitro demonstrated that atorvastatin reduces the matrix degradation potential of proinflammatory macrophages by reducing intracellular MMP-14 activation [75]. In patients with carotid artery stenosis, Eilenberg et al. [76] reported that circulating neutrophil gelatinase-associated lipocalin (NGAL) and MMP-9/NGAL are associated with plaque vulnerability and that statin treatment could contribute to plaque stabilization by reducing circulating NGAL and MMP-9/NGAL levels. Finally, incretin analog drugs have been also tested for therapeutic properties as modulators of atherosclerosis. Gallego-Colon et al. [77] have recently shown that Exenatide downregulates the expression of MMP-1, MMP-2, and MMP-9 in coronary artery smooth muscle cells under inflammatory conditions, suggesting a possible role of incretin analog drugs in therapy for coronary atherosclerosis.

## 5. Conclusions

MMPs and TIMPs are the main actors in the ECM remodeling, and their behavior is altered in pathological states. The imbalance between the synthesis and degradation of ECM components underlies the development of many diseases. The controversial results reported up to this moment could be consequences of the pattern of expression and activity of MMPs and their inhibitors, which depend on the stage of the diseases.

Targeting the ECM as a potential therapeutic option should involve MMPs and TIMPs modulation. A deeper understanding of the MMPs behavior in ECM biology must be attained to uncover new targets for future therapies in high incidence pathologies such as obesity and hepatic and cardiovascular diseases.
